# Supplementary material for: Contrasting long-term temperature trends reveal minor changes in projected potential evapotranspiration in the US Midwest
Source: Nat Commun. 2021 Mar 5;12:1476. doi: 10.1038/s41467-021-21763-7 (PMC7936007; doi:10.1038/s41467-021-21763-7)
Supplement: Supplementary file 1 — Supplementary Information [file 41467_2021_21763_MOESM1_ESM.pdf]

Contrasting long-term temperature trends reveal minor changes in projected potential evapotranspiration in the US Midwest

Bruno Basso[1,2*], Rafael Martinez-Feria[1], Lydia Rill[1] and Joe T. Ritchie[1]

[1] Department of Earth and Environmental Sciences, Michigan State University, East Lansing, MI, USA; [2] W.K. Kellogg Biological Station, Michigan State University, Hickory Corners, MI, USA

*Corresponding author email: basso@msu.edu

Supplementary Information

Supplementary Figure 1. Absolute and relative changes in vapor pressure deficit (VPD) projected by 2050

Supplementary Figure 2. Propagated uncertainty in vapor pressure deficit (VPD) projections into 2050.

Supplementary Figure 3. Assessment of the validity of simulated crop yields.

Supplementary Figure 4. Comparison of ΔPET projections.

Supplementary Figure 5. Impacts of projected summer temperature trends on potential evapotranspiration as simulated by the SALUS model.

Supplementary Figure 6. Five-year summaries of the JJA climate record across all NWS-COOP stations.

Supplementary Figure 7. Location of stations from the Automated Surface Observing System (ASOS) network

Supplementary Figure 8. Generalized Additive Model (GAM) for prediction of average daily dewpoint temperature (Tdew) for the JJA period.

Supplementary Figure 9. Prediction of Tdew at the testing ASOS stations.

Supplementary Figure 10. Exploratory analysis for times-series selection

Supplementary Figure 11. Distribution of the lag-1 autocorrelation of the detrended seasonal temperature data at the NWS-COOP stations for the three time series.

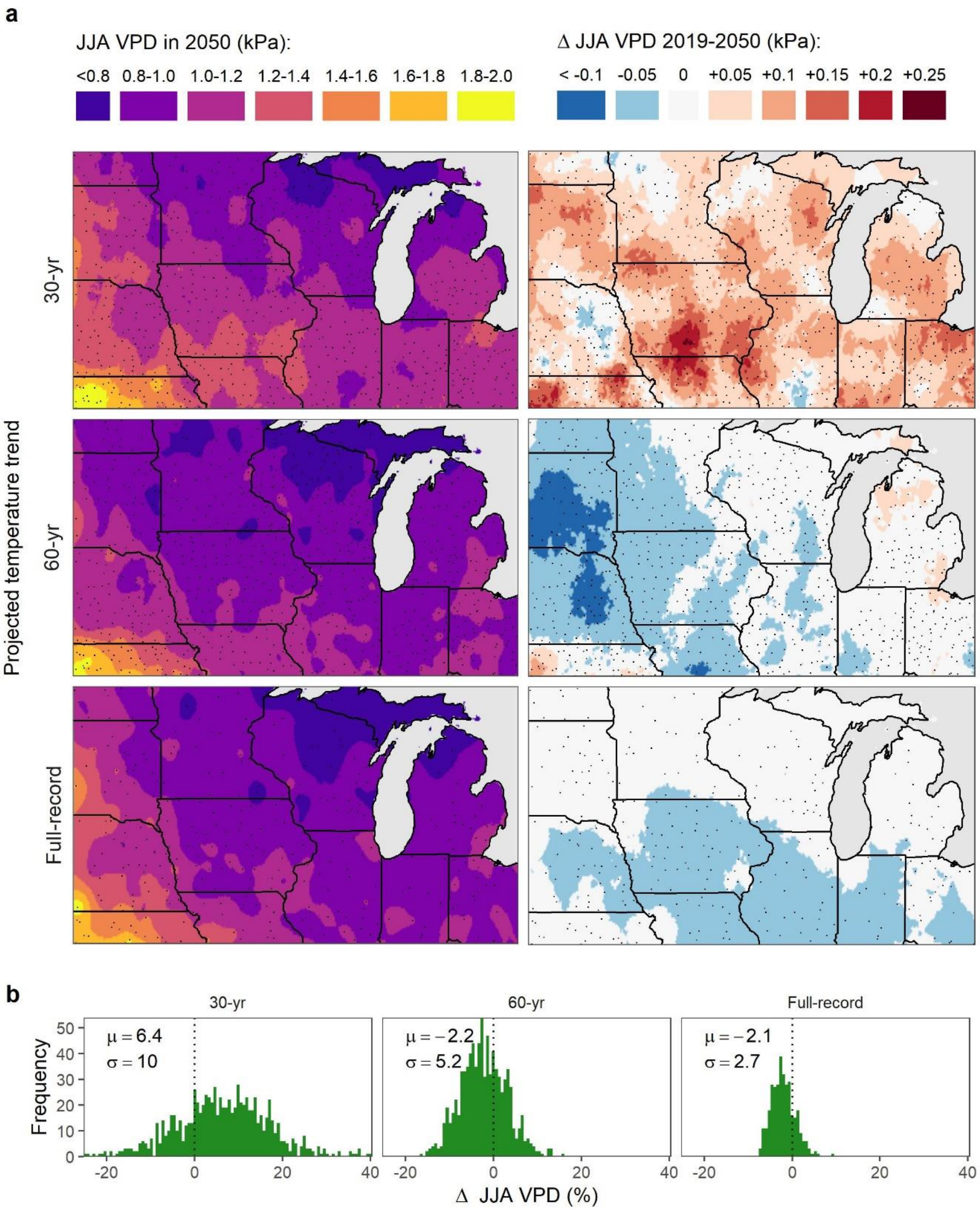

Supplementary Figure 1. Absolute (a) and relative (b) changes in vapor pressure deficit (VPD) projected by 2050. These are based on temperature trends for 30-yr, 60-yr and full-record times series.

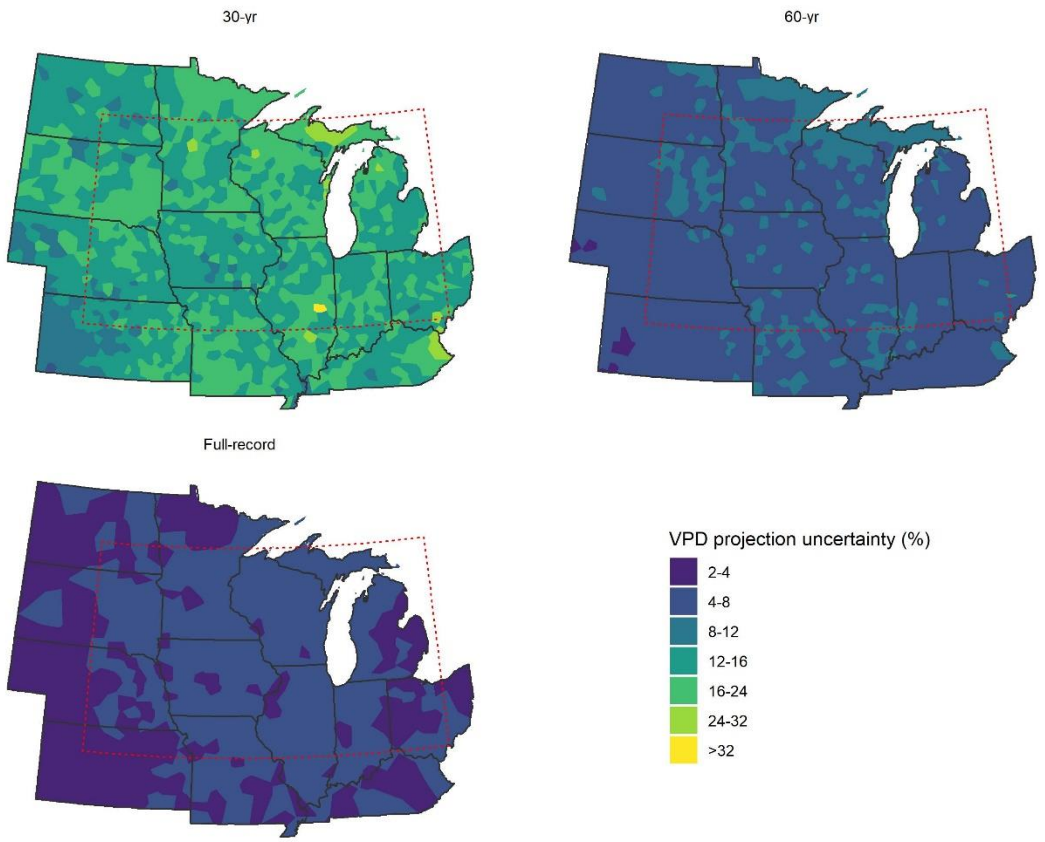

Supplementary Figure 2. Propagated uncertainty in vapor pressure deficit (VPD) projections into 2050. These were derived from the computed standard errors of the temperature trends for 30-yr, 60-yr and full-record times series.

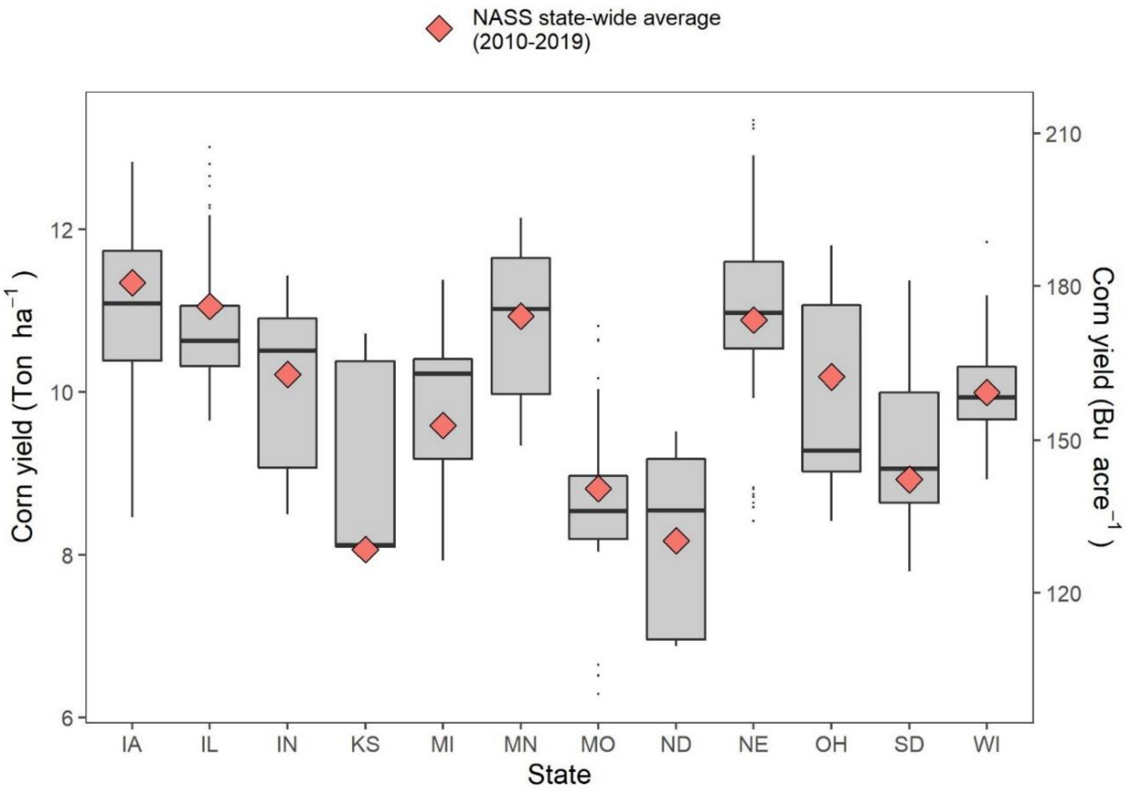

Supplementary Figure 3. Assessment of the validity of simulated crop yields. The simulated corn yields at the weather stations (quantile boxes) are compared against the state-aggregated yields reported by the National Agricultural Statistics Service (NASS) for the 2010-2019 period (diamonds).

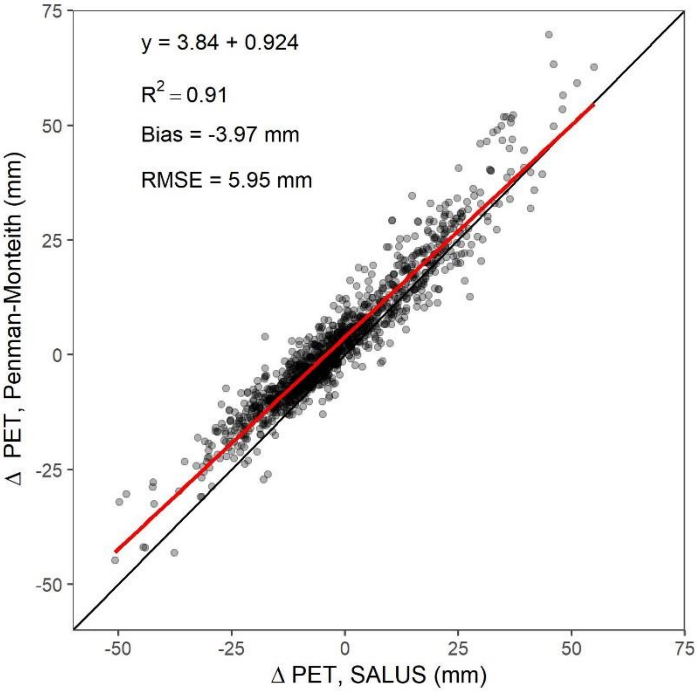

Supplementary Figure 4. Comparison of ΔPET projections. The x-axis shows the simulated ΔPET by the SALUS model and the y-axis is the ΔPET estimated by the offline calculation using the Penman-Monteith approach.

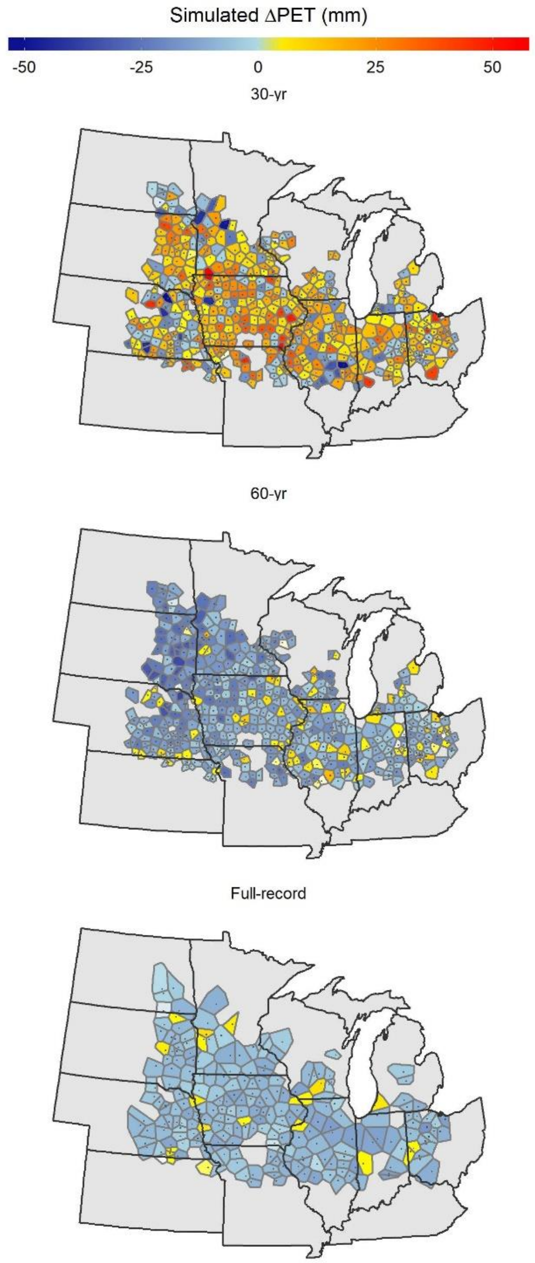

Supplementary Figure 5. Impacts of projected summer temperature trends on potential evapotranspiration as simulated by the SALUS model. Voronoi polygons indicate coverage of stations within major corn-producing Midwestern counties.

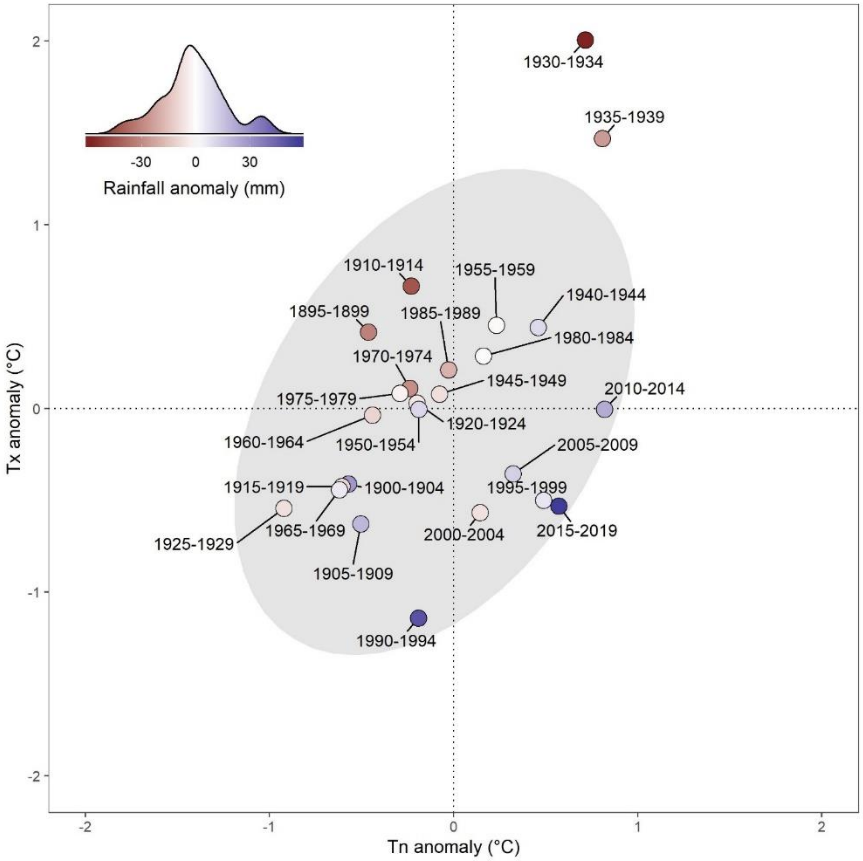

Supplementary Figure 6. Five-year summaries of the JJA climate record across all NWS-COOP stations. The shaded ellipse indicates the 2D normal distribution at the 95% level.

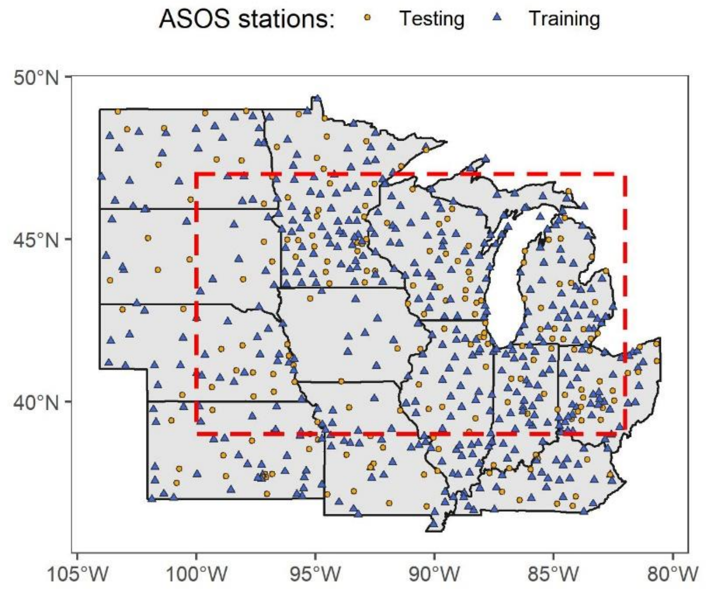

Supplementary Figure 7. Location of stations from the Automated Surface Observing System (ASOS) network. These data were (n=660) used for modeling average daily dewpoint temperatures. Stations were divided with a random 70-30% split for training and testing purposes, respectively.

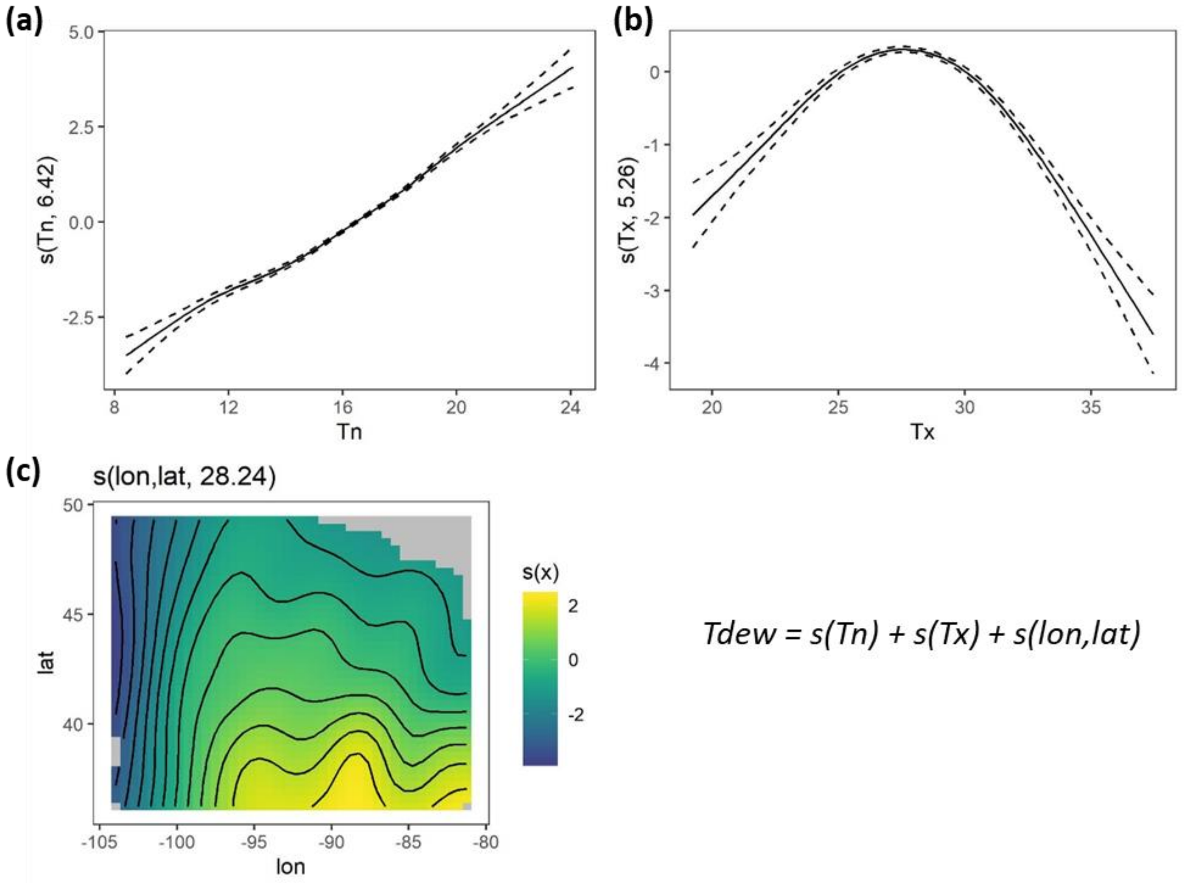

Supplementary Figure 8. Generalized Additive Model (GAM) for prediction of average daily dewpoint temperature (Tdew) for the JJA period. The GAM was fitted with data from the training ASOS stations, using as predictors the smooth effects of average daily minimum temperature (Tn), average daily maximum temperature (Tn), and the 2D smooth effect of longitude and latitude.

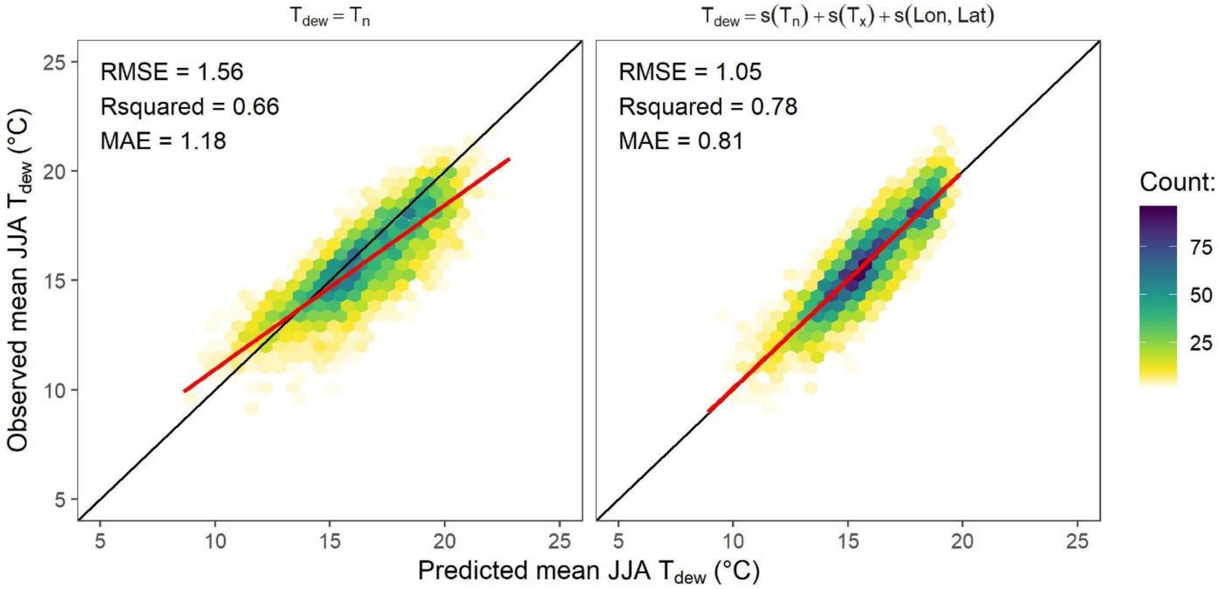

Supplementary Figure 9. Prediction of T_{dew} at the testing ASOS stations. The prediction using the developed GAM performed better (R^2 = 0.78) compared to using the daily minimum temperature (T_n) as a surrogate for T_{dew} (R^2 = 0.66).

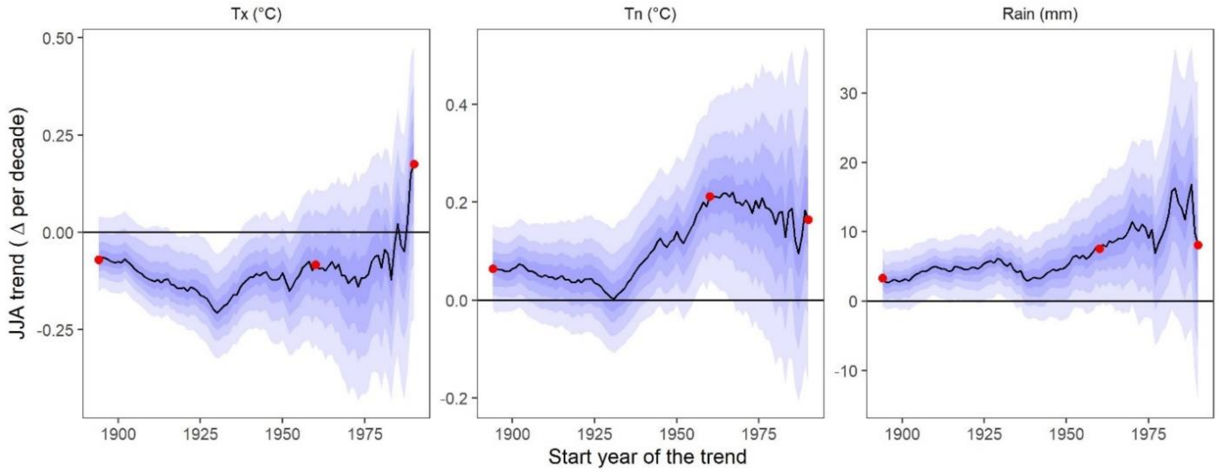

Supplementary Figure 10. Exploratory analysis for times-series selection. Solid line indicates the median value of the Sen's slope across all stations and the shade area indicate the range**.** Red symbols indicate the beginning of the selected time-series: 1894-2019, 1960-2019 and 1990-2019**.**

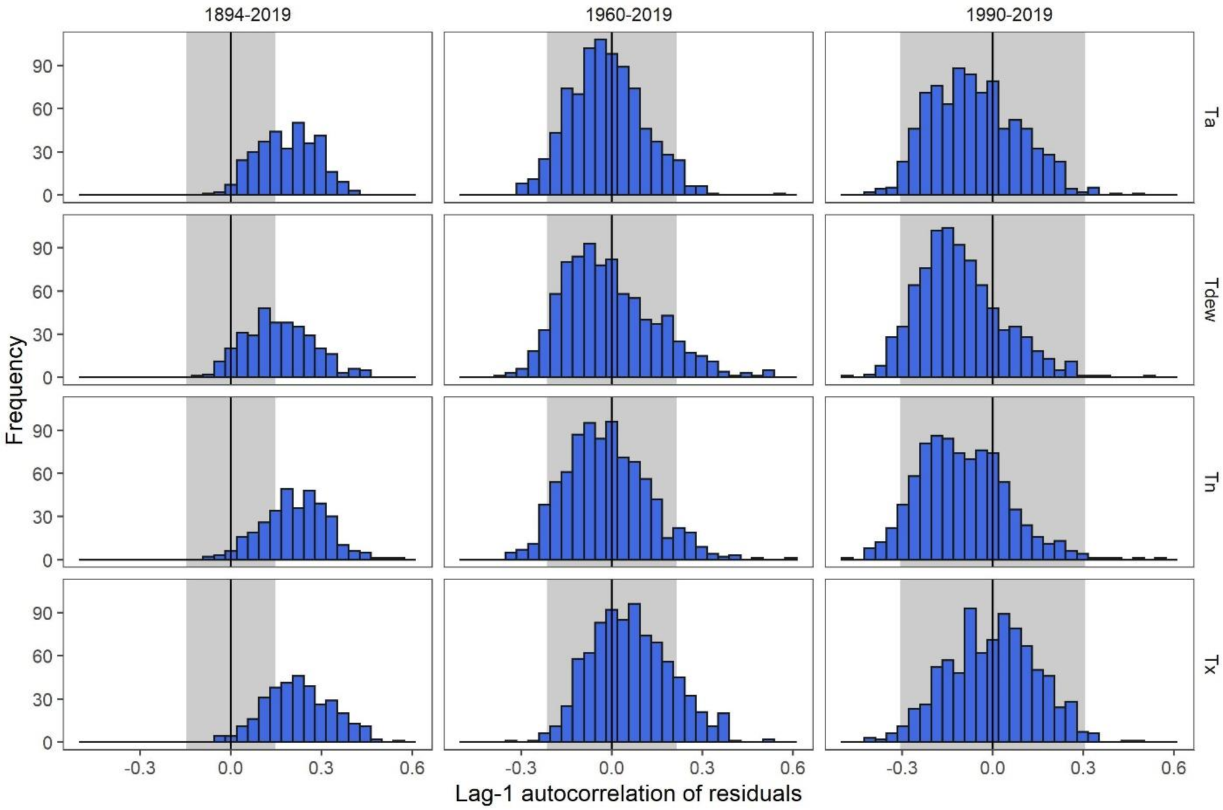

Supplementary Figure 11. Distribution of the lag-1 autocorrelation of the detrended seasonal temperature data at the NWS-COOP stations for the three time series. Shaded vertical area indicates non-significant autocorrelation at the α= 0.1 level.
